# Visualizing posttranslational and epigenetic modifications of endogenous proteins in vivo

**DOI:** 10.1007/s00418-015-1344-0

**Published:** 2015-07-03

**Authors:** Hiroshi Kimura, Yoko Hayashi-Takanaka, Timothy J. Stasevich, Yuko Sato

**Affiliations:** Graduate School of Bioscience and Biotechnology, Tokyo Institute of Technology, 4259 Nagatsuta, Midori-ku, Yokohama, 226-8501 Japan; Department of Biochemistry and Molecular Biology, Colorado State University, Campus Delivery 1870, Fort Collins, CO 80523 USA

**Keywords:** Chromatin, Epigenetics, Posttranslational protein modification, Live cell imaging

## Abstract

Protein localization and dynamics can now be visualized in living cells using the fluorescent protein fusion technique, but it is still difficult to selectively detect molecules with a specific function. As a posttranslational protein modification is often associated with a specific function, marking specifically modified protein molecules in living cells is a way to track an important fraction of protein. In the nucleus, histones are subjected to a variety of modifications such as acetylation and methylation that are associated with epigenetic gene regulation. RNA polymerase II, an enzyme that transcribes genes, is also differentially phosphorylated during the initiation and elongation of transcription. To understand the mechanism of gene regulation in vivo, we have developed methods to track histone and RNA polymerase II modifications using probes derived from modification-specific monoclonal antibodies. In Fab-based live endogenous modification labeling (FabLEM), fluorescently labeled antigen-binding fragments (Fabs) are loaded into cells. Fabs bind to target modifications in the nucleus with a binding time of a second to tens of seconds, and so the modification can be tracked without disturbing cell function. For tracking over longer periods of time or in living animals, we have also developed a genetically encoded system to express a modification-specific intracellular antibody (mintbody). Transgenic fruit fly and zebrafish that express histone H3 Lys9 acetylation-specific mintbody developed normally and remain fertile, suggesting that visualizing histone modifications in any tissue in live animals has become possible. These live cell modification tracking techniques will facilitate future studies on epigenetic regulation related to development, differentiation, and disease. Moreover, these techniques can be applied to any other protein modification, opening up new avenues in broad areas in biology and medicine.

## Introduction

Thanks to the discovery of fluorescent proteins and development of live cell microscopy technologies, any protein can now be visualized in living cells and in organisms (Shaner et al. 2005; Giepmans et al. 2006; Chalfie 2009). The expression level of the tagged protein is also adjustable to the endogenous level by inserting the tag into the endogenous gene locus by homologous recombination (Yang et al. 2013). Therefore, the changes in the protein localization can be tracked over time in single cells and also during development and differentiation in model systems. A simple localization analysis, however, cannot distinguish molecules that harbor specific function from non-functional ones. For example, only a fraction of RNA polymerase II (RNAP2) molecules are transcribing genes at a given time, while the other molecules may be freely diffusing in the nucleus or undergoing repeat binding and unbinding with DNA until they become assembled into preinitiation complexes (Kimura et al. 1999, 2002; Stasevich and McNally 2011). Therefore, one of the next challenges in live cell microscopy is to selectively detect protein molecules with specific functions, such as enzymatically active molecules and ones in specific protein complexes. By using technologies that can measure protein mobility, including fluorescence recovery after photobleaching (FRAP), fluorescence correlation spectroscopy and single-molecule tracking, different kinetic fractions (e.g., stable DNA-bound and DNA-unbound forms; and multimeric and monomeric forms) can be detected (Mazza et al. 2012). A few different kinetic fractions have successfully been separated by these techniques combined with kinetic modeling and/or simulation, but rigorous analyses are required to separate different fractions with similar kinetics and the interpretation relies on the models (Darzacq et al. 2007; Stasevich and McNally 2011). Therefore, it would be ideal if a specific mark for functional molecules could be detected in living cells. One strategy for detecting a specific form is to monitor a protein–protein interaction, as many proteins exhibit distinct functions in different protein complexes with specific partners. For this purpose, one can use Förster/fluorescence resonance energy transfer (FRET), which occurs between two spectrally overlapping donor and acceptor fluorophores when they are close enough and in the right orientation (Sekar and Periasamy 2003). In practice, however, intermolecular FRET is quite difficult to detect, and only limited cases are available (Aoki et al. 2012). Another strategy is to label a posttranslational modification, as many proteins are subjected to a variety of modifications and some are associated with protein function. Indeed, antibodies directed against specific modifications have in fact been widely used to detect functional and/or specific forms of proteins. However, it has only recently become possible to use modification-specific antibodies for live cell imaging (Hayashi-Takanaka et al. 2009; Kimura et al. 2010).

**Fig. 1:**
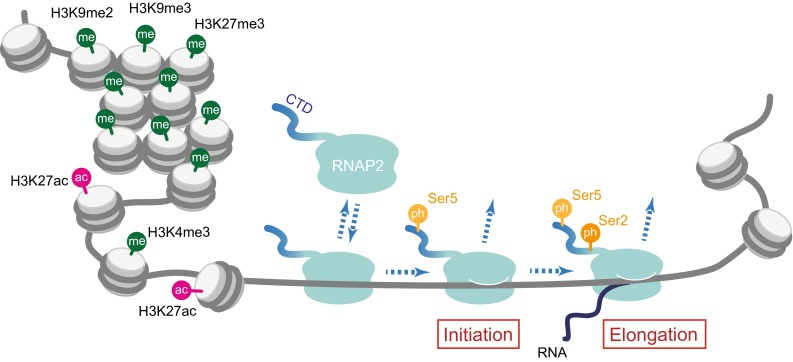
Schematic illustration of histone and RNAP2 modifications. Transcriptionally inactive condensed chromatin harbors inactive histone marks, such as H3K9me2, H3K9me3, and H3K27me3. In contrast, transcriptionally active promoter regions are associated with active marks, such as H3K4me3 and H3K27ac. RNAP2 phosphorylation is associated with the transcription cycle. Ser5 and Ser2 along the Rpb1-CTD repeat are phosphorylated during the initiation and elongation, respectively

Posttranslational protein modification plays a critical role in gene regulation, as it does in many other important biological events, such as signal transduction. Both the template chromatin for transcription and the transcribing enzyme, RNA polymerase, can be heavily modified (Fig. 1) (Bannister and Kouzarides 2011; Eick and Geyer 2013; Kimura 2013; Jeronimo et al. 2013), and our approach to tracking their modifications in living cells has been a breakthrough to fully understand how genes are regulated in vivo.

## Histone modification and gene regulation

In eukaryotic cell nuclei, genomic DNA is packed into chromatin. The fundamental unit of chromatin is called the “nucleosome,” a structure containing ~ 150 bp of DNA that is wrapped around a histone octamer (two copies of each of the core histones, H2A, H2B, H3, and H4) (Luger et al. 1997). Therefore, many important biological events that use DNA as a template and/or substrate, such as transcription, DNA replication, and DNA repair, have to deal with this structure. The nucleosome formation has been thought to have an inhibitory role in transcription, as transcription factor binding is often prevented; however, recent studies have shown that histones function both positively and negatively in the regulation of gene expression (ENCODE Project Consortium 2012). Such positive and negative regulations are largely governed by posttranslational modifications on specific amino acid residues on histones (Fig. 1). As histones, particularly H3 and H4, bind quite stably to DNA (Kimura and Cook 2001; Kimura 2005), their posttranslational modifications can be inheritable epigenetic marks, in addition to direct DNA modifications, which are of limited diversity, such as methylation and hydroxymethylation on the 5-position at the cytosine base (Smith and Meissner 2013). In contrast, histones are subjected to a variety of modifications, including acetylation, methylation, phosphorylation, and ubiquitylation, on many different amino acid residues. Among the various modifications on four core histones, acetylation and methylation on specific lysine residues on H3 appear to be most relevant to gene regulation (Bannister and Kouzarides 2011; Kimura 2013). On a lysine residue, the primary amine is positively charged, and when single acetylation occurs at the amino group, the charge is neutralized. This neutralization could loosen the contact between negatively charged DNA and lysine-rich histones so that nucleosomal DNA may become more accessible to transcription factors. Such loosening can be observed by biochemical analysis when massive or a critical acetylation occurs (Brower-Toland et al. 2005; Di Cerbo et al. 2014), but the information of individual acetylation is also transmitted through a “reader” protein that binds to a specific acetyl lysine (Yun et al. 2011). Methylation on lysine can also occur on the primary amine. As each of the three hydrogen on the primary amine can be replaced by a methyl group without changing the positive charge, three different levels of methylation, monomethylation (me1), dimethylation (me2), and trimethylation (me3), are possible. A number of proteins that bind specifically to methyl lysine are also present (Yun et al. 2011). Thus, a single lysine residue could have five different modification states (i.e., acetyl, unmodified, me1, me2, and me3), and specific binding proteins can recognize these specific modifications.

Histone H3 is acetylated at K9, K14, K18, K23, and K27, and all acetylation is essentially correlated with transcriptional activation. Among them, H3K27 acetylation (H3K27ac) is specifically enriched in transcription start sites and enhancers of active or potentially active genes (Fig. 1) (Kimura 2013). Others, such as H3K9 acetylation (H3K9ac) and H3K14 acetylation (H3K14ac), are distributed more broadly (Karmodiya et al. 2012). The correlation of methylation on H3 with transcription activity depends on its level and the residue. Methylation of H3K4 is associated with transcriptionally active genes. In particular, trimethylated H3K4 (H3K4me3) is known to be a good marker for transcription start sites of transcribed genes (Fig. 1) (ENCODE Project Consortium 2012). In contrast, methylations on H3K9 and H3K27 are generally associated with inactive genes (Fig. 1). However, these histone modifications may not be necessarily prerequisite codes for turning genes on and off (Henikoff and Shilatifard 2011). Some could affect the transcription kinetics, and others could be added as a consequence of pioneering transcription.

Histone modifications change locally and globally during development and differentiation and in response to external stimuli. As dysregulation of histone modifications is often associated with diseases such as cancer (Portela and Esteller 2010; Butler et al. 2012; You and Jones 2012), it is important to reveal the function and regulatory mechanism of histone modifications. Whereas the genome distribution of several histone modifications has been measured in many different cell types, how these modifications are regulated at the single cell level in vivo remains largely unknown. Therefore, it has been important to develop a method to visualize histone modifications in living cells and organism.

## RNA polymerase II modification and the transcription cycle

RNA polymerase II (RNAP2) is a multi-subunit DNA-dependent RNA polymerase that transcribes most genes and some small noncoding RNA. The catalytic, largest, subunit of RNA polymerase II (Rpb1) harbors heptapeptide (YSTPSPS) repeats at the C-terminal domain (CTD) (Eick and Geyer 2013; Jeronimo et al. 2013). In human and mouse Rpb1, this heptapeptide is repeated 52 times with slight variations at the latter part of the tail, while Rpb1 in lower organisms harbors shorter repeats at the CTD (e.g., 27 times in budding yeast). All of the Ser residues in the heptapeptide (i.e., S2, S5, and S7) are known to be phosphorylated, and the phosphorylation on S2 and S5 has been well correlated with the RNAP2 transcription cycle (Fig. 1). In brief, when RNAP2 is freely diffusing and becomes assembled into the preinitiation complex on a promoter, Rpb1-CTD is unphosphorylated. During the initiation, S5 becomes phosphorylated by Cdk7-CyclinH kinase in TFIIH. When processive transcription elongation begins, S2 becomes phosphorylated by P-TEFb (the complex of Cdk9-CyclinT) and by other kinases including Cdk12 (Bowman and Kelly 2014). Upon removal of RNAP2 from the DNA template after the completion of transcription, Rpb1-CTD becomes dephosphorylated again. Thus, the phosphorylation states of Rpb1-CTD can be well-defined markers for specific forms.

## Modification-specific monoclonal antibodies

To analyze protein modifications, specific antibodies have proven very useful (Kimura 2013; Goto and Inagaki 2014). The presence of the target modifications can be detected in situ and in cell extracts by immunochemical techniques, such as immunofluorescence, immunoblotting, and immunoprecipitation. In the early 2000s, we began to analyze histone modifications using polyclonal histone modification-specific antibodies obtained from commercial sources. However, we had been often frustrated due to the uncertainty of specificity and lot-to-lot differences. When using different antibody lots with the same catalog number, results of chromatin immunoprecipitation (ChIP) were sometimes inconsistent. Our enzyme-linked immunosorbent assay (ELISA) using synthetic peptides that contained residue-specific modifications in fact showed that some antibody samples cross-reacted with many peptides and had little specificity (Kimura et al. 2008). We then decided to generate more reliable antibodies. Although there was an argument about whether or not monoclonals are better than polyclonals, we chose to generate monoclonals because, in principle, once good hybridoma cells are established, the properties of monoclonal antibodies should be retained over time and their supply is unlimited. Making histone modification-specific antibodies was not easy work, however, because the specific antibodies need to recognize both the difference of the single methyl group (e.g., H3K9me3 and H3K9me2) and the neighboring amino acids (e.g., H3K9me3 and H3K27me3) (Kimura 2013). After spending lots of time and effort to screen specific clones (e.g., by ELISA using 46 different peptides, immunoblotting, immunofluorescence, and ChIP), we have made a panel of specific monoclonal antibodies directed against histone H3 and H4 modifications (Kimura et al. 2008; Hayashi-Takanaka et al. 2009, 2011, 2014; Rechtsteiner et al. 2010; Chandra et al. 2012). These antibodies have been used for western blotting, immunofluorescence and chromatin immunoprecipitation in many different laboratories, including those involved in the modENCODE project (Egelhofer et al. 2011) and the International Human Epigenome Project. We have also generated monoclonal antibodies directed against unphosphorylated and phosphorylated Rpb1-CTD of RNAP2. Importantly, these antibodies are also applicable to immunohistochemistry using human patient specimen (Yokoyama et al. 2013, 2014) and immunofluorescence using mouse tissue sections (Eberhart et al. 2012, 2013; Kamiunten et al. 2014).

## Fab-based live endogenous modification labeling

We thought that the monoclonal antibodies we had generated might be useful for detecting the target modifications in living cells. For that, we first labeled a native antibody (as IgG form) with a fluorescent dye and loaded it into cells. However, this initial attempt using the whole IgG did not work well; cytoplasmically loaded IgG did not enter the nucleus because it is bigger than the nuclear pore (Hayashi-Takanaka et al. 2011). Even though IgG became accessible to chromatin during mitosis and entered the nucleus after cell division, its high affinity and bivalency could cause toxic effects to cells by blocking the target.

**Fig. 2:**
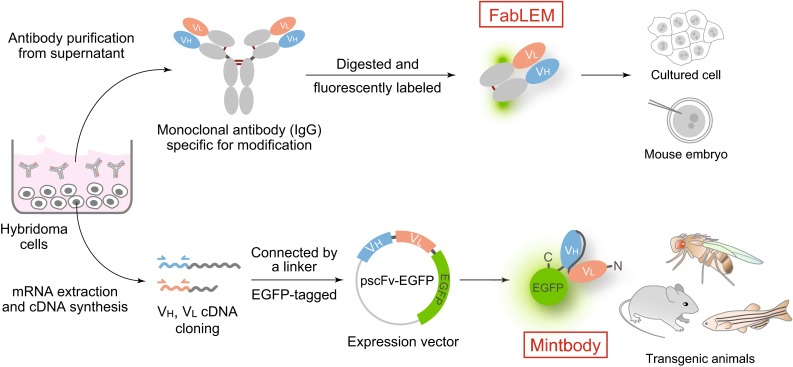
Schematic illustration of FabLEM and mintbody methods. Once hybridoma cells that produce specific antibody directed against a modification site are generated, two methods for tracking the modification in living cells are possible. (*Top*) FabLEM: Modification-specific antibody is purified from hybridoma cell culture supernatant, and fluorescently conjugated Fab fragments are prepared for loading into cells or injecting into mouse embryos. (*Bottom*) Mintbody: cDNA encoding the variable regions of heavy (V_H_) and light chains (V_L_) can be cloned into a vector to be expressed as an scFv fused with the green fluorescent protein. The expression vector can be transfected into cells or embryos to generate transgenic lines

**Fig. 3:**
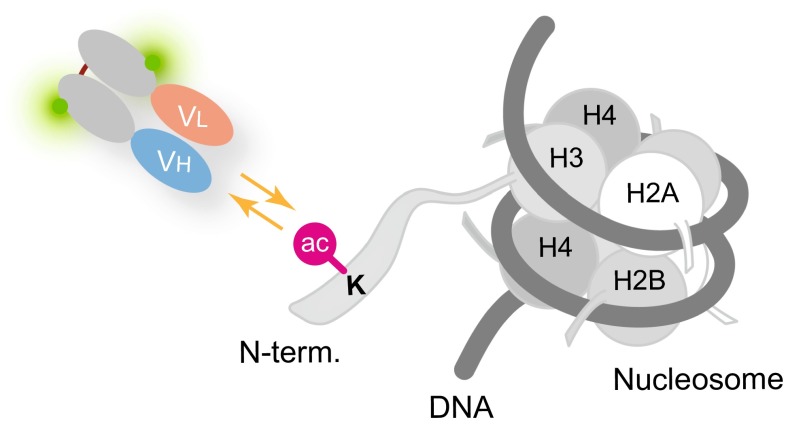
Transient binding of Fab to target modifications in living cells. Fluorescently labeled Fab molecules that entered the nucleus repeatedly bind and unbind to target modifications (e.g., acetylated histone H3). The localization of the modification can therefore be detected by Fab concentration. The transient binding of Fabs allows the access of endogenous proteins to the modification, and thus, Fabs have little effects on cell physiology

In contrast to the whole IgG, fluorescently labeled antigen-binding fragments (Fabs) were suitable for detecting the endogenous histone modifications (Hayashi-Takanaka et al. 2009, 2011). We prepared Fab fragments from the whole IgG by protease digestion and labeled these with an amine-reactive fluorescent dye, before loading into living cells (Fig. 2, top). After loaded into cells, Fabs diffuse throughout the cytoplasm and enter the nucleus through the pore by diffusion, as their size (~50–60 kD) was just smaller than the pore size. If the target modification is present in the nucleus, Fabs bind to the target and become concentrated in the nucleus (Fig. 3). Fabs could bind to the target modification stably and block the access of cellular factors to the modification; however, Fab binding was transient and did not interfere with cell division. FRAP revealed that the binding time of Fabs to the target modifications in living cell nuclei was in a range from <1 to ~30 s, depending on the binding affinity. This might be unexpected because antibody binding to the epitope is thought to be quite stable. Unlike whole IgG that has a bivalent antigen-binding site, Fabs are monovalent and the binding affinity is much lower. In addition, the high macromolecule concentration (>100 mg/ml) in living cells can cause more frequent molecular collisions to force the dissociation of bound proteins. Indeed, Fabs loaded into cells at ~1 µM did not disturb cell growth. Furthermore, mouse preimplantation embryos injected with Fab developed normally to birth. It is thus concluded that the endogenous modifications can be visualized by using fluorescently labeled Fab without affecting cell function. We named this technique Fab-based live endogenous modification labeling, or FabLEM (Hayashi-Takanaka et al. 2011).

**Fig. 4:**
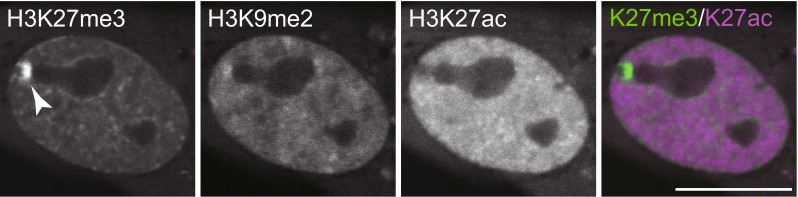
Histone modifications in living cells detected by FabLEM. Three different Fabs (Alexa Fluor 488-labeled anti-H3K27me3, Cy3-labeled anti-H3K9me2, and Cy5-labeled anti-H3K27ac) are loaded into human hTERT-RPE1 (telomerase-immortalized retina pigment epithelial) cells. Fluorescence images in living cells were collected using a confocal microscope. H3K27me3-specific Fab, plus H3K9me2-specific Fab to some extent, is concentrated on the inactive X chromosome (*arrowhead*). H3K27ac-specific Fab is excluded from the inactive X chromosome. *Bar* 10 μm

By using FabLEM, simultaneous detection of multiple modifications is possible, as fluorescent dyes are directly conjugated to Fabs. In principle, any fluorescent dye can be conjugated to Fabs, but some dyes interfere with Fab activity and/or tend to cause cytoplasmic aggregation. A systematic screening has revealed that Alexa Fluor 488, Cy3, and Cy5 or CF640 are the most suitable conjugation partners with Fabs among green, red, and far-red fluorescence dyes, respectively (Hayashi-Takanaka et al. 2014). By using multiple Fabs labeled with these dyes, three different histone modifications can be visualized simultaneously in single cells (Fig. 4). Once loaded into cells, Fab molecules are quite stable, and their localization can be followed for a few days until the signal becomes too diluted by cell division or Fabs are trapped in cytoplasmic vesicles.

FabLEM has been useful to track both the localization of modifications and the global modification level in the nucleus. For example, the concentration of H3K27me3 on inactive X chromosomes can be visualized in living cells (Fig. 4), which allowed us to track the intranuclear localization and replication timing of inactive X chromosomes in living cells (Hayashi-Takanaka et al. 2011). The changes in global modification levels can be monitored by FRAP or by comparing the nuclear/cytoplasmic intensity ratio measurements. When the modification level is increased, more Fab molecules become bound to chromatin, and so the difference in bound fraction and/or on-rate can be measured by FRAP. In addition, as unbound Fabs can diffuse into the cytoplasm, the ratio of nuclear and cytoplasmic fluorescence intensity is correlated with the bound and unbound fractions. Therefore, the changes can be detected by simple intensity ratio measurements. In these ways, we have been able to measure the effect of histone deacetylase inhibitor on histone H3 acetylation and methylation in a sensitive manner.

## Modification-specific intracellular antibody

**Fig. 5:**
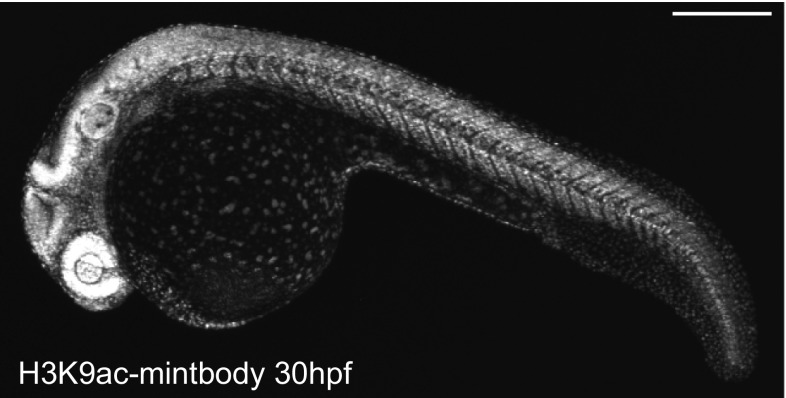
H3K9-mintbody in zebrafish. Zebrafish expressing H3K9-mintbody developed normally. A fluorescence image of a zebrafish expressing H3K9-mintbody (30 h post fertilization) was collected using a confocal microscope. *Bar* 100 μm

Although FabLEM is a powerful technique, it requires purified Fabs and direct loading into cells, which may prevent long-term and high-throughput assays. In vivo analysis using model organisms is also limited except just after fertilization during which microinjection and imaging are relatively easy. To overcome these limitations, we have also developed a genetically encoded system using single-chain variable fragments (scFv; Ahmad et al. 2012) tagged with the enhanced green fluorescent protein (EGFP). We cloned the scFv coding sequence from hybridoma cells producing the specific antibody against histone H3 Lys9 acetylation (H3K9ac) and then genetically fused the scFv with EGFP (Fig. 2, bottom). We named the scFv-EGFP probe a modification-specific intracellular antibody, or “mintbody” (Sato et al. 2013). The H3K9ac-specific mintbody (H3K9ac-mintbody) bound to the target acetylation in living cells, and the changes in acetylation levels in response to a histone deacetylase inhibitor could be monitored by FRAP or the nuclear/cytoplasmic intensity ratio, just like FabLEM. Making use of the genetically encoded system, we have generated transgenic fruit fly and zebrafish that express the H3K9ac-mintbody. Importantly, those transgenic Drosophila and zebrafish developed normally and remain fertile, indicating that the expression of mintbody at a certain level does not affect development and differentiation (Fig. 5).

Although this mintbody strategy can be used for any other modification, intracellular expression of scFv is quite difficult because of misfolding and aberrant aggregation (Stocks 2005; Kvam et al. 2010). To obtain functional mintbodies, it will be necessary to screen scFv from several different hybridoma clones and/or to introduce mutations to improve the folding and stability in the cytoplasm, as a few amino acid substitutions can affect the functionality of mintbody (Sato et al. 2013).

## Histone modification and transcription activation dynamics revealed by FabLEM and mintbody

**Fig. 6:**
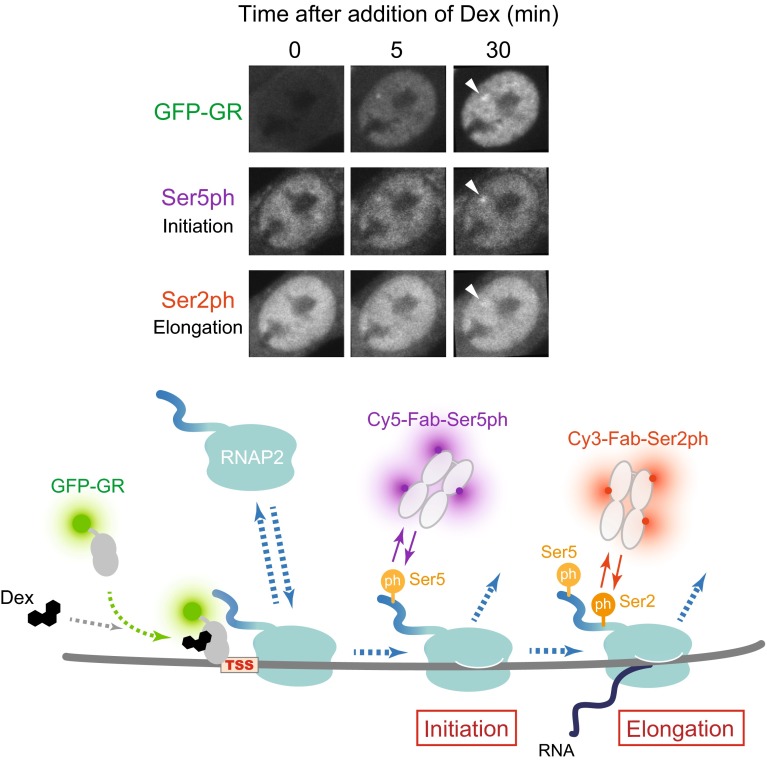
RNAP2 activation kinetics revealed by FabLEM. Fluorescence images (*top*) and schematic illustration (*bottom*) showing how RNAP2 activation by FabLEM is tracked. After addition of dexamethasone (Dex), a steroid hormone that binds to the glucocorticoid receptor (GR), to cell culture medium, GFP-GR enters the nucleus and accumulates at the gene array (*top, arrowheads*). FabLEM using RNAP2 phosphorylation-specific Fabs (Cy5-labeled Rpb1-CTD-Ser5ph and Cy3-labeled Rpb1-CTD-Ser2ph) revealed the timing of initiation and elongation of RNAP2 at the array

We have recently applied the FabLEM technique to measure the kinetics of RNAP2 in living cells and to quantify the effects of histone modifications on transcription (Stasevich et al. 2014a, b). As a model system, we used a mouse cell line that stably expresses GFP-tagged glucocorticoid receptor (GFP-GR) and harbors a genome-integrated gene array consisting of ~200 copies of glucocorticoid-responsive promoter (the mouse mammary tumor virus long-terminal repeat). Transcription of the gene array can be activated by addition of glucocorticoid hormone, which induces nuclear translocation of GR for gene activation. After loading Cy3- and Cy5-labeled Fabs that recognize differentially phosphorylated forms of RNAP2 and treating cells with the hormone, the kinetics of RNAP2 recruitment (unphosphorylated), initiation (S5 phosphorylated), and elongation (S2 phosphorylated) at the array after GFP-GR accumulation were determined (Fig. 6). Quantitative measurements and fitting to mathematical models revealed that the transition from initiation to elongation is quite efficient at the array. This high elongation efficiency was correlated with the level of preexisting histone H3K27ac, which appeared to be controlled by a balance between p300 histone acetyl transferase and histone deacetylase 4 or 7. Thus, H3K27ac can alter downstream transcription kinetics by indirectly recruiting P-TEFb kinase, in addition to enhancing the binding of GFP-GR. This study indicates a mechanism for how a histone modification can contribute to the regulation of transcription by RNAP2 in living cells (Stasevich et al. 2014a).

A correlation between histone H3 acetylation and gene activation in vivo has also been revealed using Drosophila expressing H3K9ac-mintbody. In Drosophila very early embryos, H3K9ac-mintbodies distributed nearly homogenously, but became concentrated in nuclei when zygotic gene activation occurs (Sato et al. 2013). This is consistent with H3 acetylation serving as a positive mark for transcriptional activation.

## Concluding remarks

We anticipate that the techniques we have developed (FabLEM and mintbody) will be valuable tools for understanding the dynamics of histone modifications and transcription in both cell cultures and in vivo. In particular, developing a panel of mintbody probes specific to various modifications should be encouraged, as the genetically encoded system is versatile and convenient to use. In addition, cells expressing mintbodies will be useful for screening and evaluating potential diagnostic inhibitors that affect epigenetic modifications. While other techniques such as FRET-based sensors can also monitor epigenetic regulation in living cells, in particular the balance between the modifying and demodifying enzymes, they cannot quantify the endogenous modifications themselves even though they seem to be suitable for assaying the effects of inhibitors (Sasaki et al. 2012). Importantly, our methodology can be applied to any other protein modification, opening up new avenues in broad areas in biology and medicine.
